# Development of a clinical investigation protocol for occupational auditory health

**DOI:** 10.47626/1679-4435-2023-1189

**Published:** 2024-08-05

**Authors:** Rubens Jonatha dos Santos Ferreira, Julia Lujan Pichamoni, Camila Nascimento Monteiro, Ana Carolina Cintra Nunes Mafra, Ana Loísa de Lima e Silva Araújo, Marine Raquel Diniz da Rosa

**Affiliations:** 1 Saúde Populacional, Hospital Sírio-Libanês, São Paulo, SP, Brazil; 2 Departamento de Fonoaudiologia, Universidade Federal da Paraíba, João Pessoa, PB, Brazil

**Keywords:** occupational health, hearing loss, noise-induced, medical history taking, saúde ocupacional, perda auditiva provocada por ruído, anamnese

## Abstract

**Introduction:**

Considering that noise is present in different work environments, occupational health regulations have been created that advocate for the care of employees’ auditory system in these environments. Occupational hearing assessment should be performed by audiologists through audiological examinations, otoscopy, as well as an interview to assess possible risk factors for the development of hearing loss. However, up to the present moment, a standardized set of updated questions for this interview has not been defined.

**Objectives:**

To develop a clinical investigation instrument for occupational auditory health that provides support for clinical decision-making and differential diagnosis.

**Methods:**

The study was conducted using Design Thinking as a methodological approach in its stages of inspiration (problem identification), ideation (theoretical foundation and protocol design), and prototyping (protocol construction).

**Experience report:**

This study was conducted with the objective of providing support for clinical decision-making and differential diagnosis of the auditory aspects of the assisted population. The Protocolo de Investigação Clínica da Saúde Auditiva Ocupacional was developed, consisting of six main sections that address medical history, lifestyle habits, exposure to non-occupational noise, work history, extra-auditory symptoms, and auditory and vestibular signs and symptoms, aimed at investigating workers’ auditory health and related aspects.

**Conclusions:**

The developed instrument can be used for data collection and assist audiologists in the occupational health teams in diagnosis and decision-making processes.

## Abstract (pt)

**Introdução:**

Considerando que o ruído está presente em diferentes ambientes laborais, foram criadas normas regulamentadoras de saúde ocupacional que preconizam o cuidado com o sistema auditivo dos colaboradores destes ambientes. A avaliação auditiva ocupacional deve ser realizada pelo fonoaudiólogo através dos exames de audiometria e meatoscopia, além de uma entrevista para avaliar possíveis fatores de risco para o desenvolvimento de perdas auditivas. Entretanto, até o presente momento não foi definido um padrão de perguntas atualizado para esta entrevista.

**Objetivos:**

Desenvolver um instrumento de investigação clínica da saúde auditiva ocupacional que ofereça suporte para tomadas de decisões clínicas e diagnóstico diferencial.

**Métodos:**

O estudo foi desenvolvido utilizando o *design thinking* como abordagem metodológica em suas etapas de inspiração (observada a problemática), ideação (fundamentação e delineamento teórico do protocolo) e prototipação (construção do protocolo).

**Relato da experiência:**

Este estudo foi realizado objetivando oferecer suporte para tomadas de decisões clínicas e diagnóstico diferencial dos aspectos auditivos da população assistida. Foi desenvolvido o Protocolo de Investigação Clínica da Saúde Auditiva Ocupacional, composto de seis seções principais que abordam o histórico clínico, hábitos de vida, exposição a ruído extraocupacional, histórico laboral, sintomas extra-auditivos e sinais e sintomas auditivos e vestibulares, que visam investigar a saúde auditiva do trabalhador e aspectos relacionados a ela.

**Conclusões:**

O instrumento desenvolvido poderá servir para a coleta de dados e auxílio para diagnóstico e tomada de decisões dos fonoaudiólogos das equipes de saúde ocupacional.

## INTRODUCTION

Work environments have been changing since the first industrial revolution. However, as a reflection of this historical movement, professionals are still working long hours, accumulating workloads, precarious working conditions, among other factors that directly affect their relationship with work, health, and quality of life.^1^ In view of the need for intervention for this population, occupational health in Brazil has made several advances, having emerged from the 1960s-1970s, following the changes in the domestic public health and preventive medicine scenario.^2^

Occupational health is an issue that has been drawing more attention in recent years, given that this profession is essential to the economy of states, covering ergonomic aspects, physical, chemical and biological risks, and environmental aspects.^3^ This means occupational health should be implemented in workplaces to promote health and prevent health problems.

Noise is one of the risk factors for occupational health. Noise can be defined as sound coming from a sound source, which acoustically shows a lack of harmonic relationships between frequencies and their components, and a deficit in the periodicity of its waves.^4^ According to Regulatory Standard No. 15 (NR-15),^5^ workers exposed to continuous or intermittent noise from their work environment are required to comply with the maximum exposure time according to the sound pressure level for each area. They should also use individual hearing protection equipment in situations where there is a serious and imminent risk of hearing loss. In addition, Regulatory Standard No. 7 (NR-7)^6^ lists tonal threshold audiometry as the main test for monitoring occupational hearing health. Therefore, occupational hearing health is an important part of the Programa de Controle Médico da Saúde Ocupacional (PCMSO - Occupational Health Medical Control Program).

Audiometry is a common test to diagnose hearing loss and indicate possible causes of hearing loss. As such, the speech and language therapist is part of the occupational health and safety team and is responsible for conducting and monitoring occupational hearing tests and the Hearing Conservation Program (HCP).^7^

In order to provide detailed occupational hearing monitoring, speech and language therapists should, before conducting audiometric tests, screen for possible factors that could trigger hearing loss, thus making up the differential diagnosis of noiseinduced hearing loss (NIHL).^8^ Although a manual for occupational anamnesis exists to standardize data collection at a nationwide level, domestic literature is lacking when it comes to standardizing instruments for mapping occupational hearing health.^9^ Therefore, this study aims to report on the experience of developing a clinical investigation tool for occupational hearing health to support clinical decision-making and differential diagnosis.

## METHODS

This study was conducted at the Saúde Populacional Sírio-Libanês, aiming to provide support for clinical decision-making and differential diagnosis of the hearing aspects of the assisted population.

**Figure 1:**
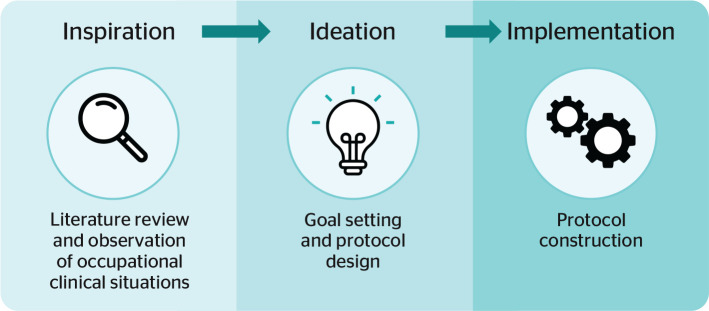
Method development of the protocol. Source: adapted from Brown.^11^

Design thinking, which is a problem-solving innovation tool,^10^ was used to develop the protocol.^10,11^ It presents three main stages to develop innovation^11^: inspiration, ideation, and implementation. This study utilized these stages (Figure 1).

### INSPIRATION

Theoretical references were searched for in domestic and international databases from January to March 2023, to provide a legal and scientific basis for the protocol on occupational exposure to noise and related factors. In addition, occupational clinical observations have analized problems that speech and language therapists who specialize in this area have experienced.

Also, SciELO, PubMed, and Google Scholar were searched for protocols covering occupational hearing issues with the same content as the protocol to be developed.

### IDEATION

The material collected previously served as the basis for thinking. This stage raised questions about the problem:

“What are the main disadvantages of occupational exposure to noise?”

“How can occupational audiological assessment be streamlined, facilitated, and subsidized?”

These questions allowed us to outline the main objectives of the protocol so that the occupational audiological assessment could be conducted concisely and effectively, considering occupational and nonoccupational factors, and previous history of risk factors for hearing loss. Soon after ideas were aligned, the goals and outline of the protocol were stipulated, clearly outlining its purposes and manner of use, so that it could be developed more objectively.

### IMPLEMENTATION

In implementation, the protocol features were introduced into concrete activity. At this stage, the structure of the protocol was developed, adding questions according to the findings selected in the literature and clinical evidence. Also, the necessary information was provided to screen for occupational and nonoccupational predisposing factors that could trigger hearing loss in employees.

## EXPERIENCE REPORT

Continuous monitoring and evaluation of occupational health indicators, which are present in a variety of work scenarios and have an impact on human body systems, especially the auditory and vestibular systems, is essential. Therefore, this study reports on the experience of the theoretical development of this protocol, which is a key stage in supporting actions to validate the content and construct of the instrument.

The investigation of occupational hearing health and related aspects should include the various factors of employees occupational and daily life activities, aiming for a comprehensive and detailed assessment of exogenous and endogenous elements that may be linked to this area.^12^ To this end, the Protocolo de Investigação Clínica da Saúde Auditiva Ocupacional (InCliSAO, Protocol for the Clinical Investigation of Occupational Hearing Health) was developed, comprising 6 main sections: clinical history, lifestyle habits, exposure to nonoccupational noise, work history, nonauditory symptoms, and auditory and vestibular signs and symptoms. The speech and language therapist or occupational physician who conducts the clinical assessment can adapt the terms of this protocol according to the reality and communicative profile of the interviewee.

The clinical history (Chart 1) aims to investigate the existence of developmental and/or acquired predisposing factors that may contribute to the development of hearing pathologies. Among these factors, perinatal infections by viral microorganisms are the main causes of perinatal sensorineural hearing loss, with toxoplasmosis, rubella, cytomegalovirus, and herpes simplex being the most common causative agents, also known by the acronym TORCH.^13^ In addition, it is necessary for occupational hearing mapping to include common childhood pathologies, such as measles and mumps, since these can pose risks to hearing health.^14^

**Chart 1:** Aspects covered in the history of clinical predisposition protocol

History of clinical predisposition From childhood to present day, have you had or currently have?			
**Medical condition/manifestation**	**Yes**	**No**	**Which type, length of time, or medication used?**
Toxoplasmosis Rubella Measles Meningitis Mumps STI/STD (HIV, HPV, Syphilis). If yes, which? Herpes simplex COVID-19 infection Sickle cell anemia Diabetes Hypertension Unbalanced cholesterol and/or triglycerides Thyroid disease Kidney disease Respiratory problems Head and/or neck trauma Acoustic trauma (bomb, gunshot, etc.) Have you had head and/or neck surgery? Have you had or are you having radiotherapy and/or chemotherapy? Have you had or are you having treatment for malaria or tuberculosis? Have you taken or are you taking antibiotics or other medications?			

InCliSAO also aims to investigate systemic diseases and other pathologies that could be harmful to the auditory and vestibular systems. Among them, hypertension and diabetes stand out, as they are risk factors for triggering hearing loss and symptoms such as tinnitus. This relationship has been widely discussed and proven in the literature over the years.^15-18^

In addition, the protocol could also collect information on clinical conditions such as anemia, tuberculosis, trauma, and infections that could damage the auditory system, and treatment with ototoxic drugs or therapies with a high potential for ototoxicity. COVID-19 infection was also a possible risk factor, since studies^19-21^ have reported that people with this condition may be more susceptible to developing auditory and vestibular symptoms, and hearing loss, which requires the investigator to take a close look at them.

The lifestyle section of the protocol addresses issues related to drinking and smoking. Santana et al.^22^ studied the influence of smoking and alcoholism on young people, and found that those addicted people had worse otoacoustic emission results compared to the population without these addictions, concluding that smoking cigarettes and drinking alcohol are major risk factors for hearing loss.

It is still necessary to investigate exposure to nonoccupational noise for a complete auditory mapping. This section of InCliSAO addresses issues related to different environments and lifestyles in which noise may be present in nonoccupational activities, as shown in Chart 2.

**Chart 2:** Exposure to nonoccupational noise section of the Protocolo de Investigação Clínica da Saúde Auditiva Ocupacional (InCliSAO, Clinical Investigation of Occupational Hearing Health Protocol)

Exposure to nonoccupational noise			
**Exposure to nonoccupational noise**	**Yes**	**No**	**How long?**
Do you usually listen to loud music? Do you wear headphones? Do you attend places with high noise exposure (gyms, clubs, churches, etc)? Do you use firearms? Have you ever participated or currently participate in musical bands? Do you live near highways?			

Exposure to high levels of sound pressure is one of the main causes of acquired hearing loss. Improper use of personal stereos is one of the reasons why this number has increased.^23^ The combination of excessive use of headphones and high-intensity playback can trigger hearing loss in individuals who frequently wear them, and increase the risk of middle ear infections when these devices are shared with improper hygiene.^22,24^ In addition, Patricio^25^ points out that residents of neighborhoods near highways can experience hearing loss as a result of continuous exposure to noise from these environments.

Constant impact noise is also considered a risk factor for hearing loss. Impact noise tends to be of high intensity for a short period of time. However, continuous exposure to this type of noise, such as firearms shooting population, can cause irreversible damage to the auditory system, since this activity combines air and bone conduction.^26^

In addition to these noises, InCliSAO also includes a work history section for workers exposed to occupational noise, as described in Chart 3. This section investigates occupational factors that can trigger hearing loss, such as noise and exposure to ototoxic chemical agents.

**Chart 3:** Occupational history section of the Protocolo de Investigação Clínica da Saúde Auditiva Ocupacional (InCliSAO, Clinical Investigation of Occupational Hearing Health Protocol)

Work history			
**Occupation**	**Yes**	**No**	**How long and how often?**
Have you worked exposed to noise on a regular and permanent basis before joining this company? How long have you been exposed to noise previously? In what position have you worked exposed to noise? During this period, have you used hearing protection? Have you ever been exposed to vibration? Have you ever been exposed to chemicals? If so, for how long, to what agents/ compounds and at what intensity? How long have you been exposed to noise at the moment? Have you always worn hearing protection when exposed? Are you exposed to vibration at work? Are you exposed to chemicals at work? If so, to what agents/compounds and at what intensity?			

Exposure to noise, the use of hearing protection, and exposure to vibration and chemicals are decisive factors when screening for NIHL. Various studies^12,27,28^ point out that exposure to these risk factors without proper protection can trigger sensorineural hearing loss, which tends to progress over the years of exposure, making it extremely important to constantly monitor the hearing of the population exposed to these factors, as recommended by legal labor standards.

In addition to hearing loss, continuous exposure to these factors can trigger extra-auditory/vestibular symptoms. InCliSAO has developed a section to investigate this topic (Chart 4), since they directly affect the quality of life and occupational health.

**Chart 4:** Occupational history section of the Protocolo de Investigação Clínica da Saúde Auditiva Ocupacional (InCliSAO, Clinical Investigation of Occupational Hearing Health Protocol)

Extra-auditory/vestibular symptoms			
**Symptom**	Yes	No	**How often?**
Headache Insomnia Irritability Anxiety			

Studies show that the onset of extra-auditory/ vestibular symptoms is directly linked to longer exposure to noise and chemicals in the workplace. Depending on its intensity and exposure, noise can affect sleep, concentration, and metabolism, which can lead to organic and psychological changes.^4,29,30^ Nunes et al.^29^ reports that workers exposed to noise for long periods tend to have higher levels of anxiety compared to other populations. It is therefore necessary to investigate these symptoms to ensure that referrals and comprehensive care can be made to improve the quality of life of these workers.

In most studies,^12,27-30^ workers exposed to noise have, in addition to these symptoms, signs and symptoms of hearing and/or vestibular disorders (Chart 5).

**Chart 5:** Signs and symptoms of hearing and vestibular symptoms section of the Protocolo de Investigação Clínica da Saúde Auditiva Ocupacional (InCliSAO, Clinical Investigation of Occupational Hearing Health Protocol)

Signs and symptoms of hearing and vestibular disorders			
**Signs and symptoms**	**Yes**	**No**	**How often?**
Otalgia Otorrhea Itching Repeated otitis On childhood and/or currently) Dizziness and/or vertigo Tinnitus Ear fullness Difficulty hearing/understanding sounds or speech Sensation of hearing loss/decline Imbalances Recruitment Frequent washing Ear surgery Barotrauma Family history of deafness			

As it affects the body as a whole, hearing loss and other symptoms can also be present in the lives of people exposed to noise. After long working hours, workers exposed to noise have mainly reported tinnitus, dizziness, difficulty understanding speech, and a feeling of reduced hearing.^28-30^ The hearing care specialist responsible for these workers needs to be aware of these signs and symptoms, as they can be predictors of further hearing and/or vestibular disorders.

As a result, InCliSAO covers the main aspects related to occupational hearing health and the factors associated with it, presenting as a support tool for making clinical and labor insurance decisions.

## CONCLUSIONS

In view of the need to map factors, signs, and symptoms that could alert us to the onset of occupational hearing loss, InCliSAO was developed in this experiment to serve as a data collection tool of these workers, and to assist in diagnoses and decision-making among the professionals responsible for monitoring occupational hearing. It is therefore recommended that this instrument be used by speech and language therapists in occupational health teams to improve clinical practice. Additionally, it can be used as a tool to ensure the safety of the employee being assessed. The next steps in this study will be specialist evaluation and validation of the protocol, making up a new publication.
